# Effectiveness of Mobile App-Based Psychological Interventions for College Students: A Systematic Review of the Literature

**DOI:** 10.3389/fpsyg.2021.647606

**Published:** 2021-05-11

**Authors:** Carla Oliveira, Anabela Pereira, Paula Vagos, Catarina Nóbrega, José Gonçalves, Beatriz Afonso

**Affiliations:** ^1^Department of Education and Psychology, University of Aveiro, Aveiro, Portugal; ^2^Department of Psychology and Education, Portucalense Institute for Human Development (INPP), Universidade Portucalense, Porto, Portugal

**Keywords:** college students, mental health, mHealth, cognitive-behavioral therapy, counseling services

## Abstract

Serious mental health disorders are increasing among college students and university counseling services are often overburdened. Mobile applications for mental health have been growing exponentially in the last decade and they are emerging in university settings as a promising tool to promote and intervene in college students' mental health. Additionally, considering the recent covid-19 pandemic, mHealth interventions, due to its nature and possibilities, may play an important role in these institutions. Our main objectives are to explore mhealth interventions in universities, regarding its conceptual framework, acceptability and efficacy outcomes and understand its impact and contributions to address treatment delivery and psychological difficulties resulting from covid-19 pandemic. The literature search was conducted in scientific databases, namely, Web of Science, Pubmed, and Scopus. A search in app stores was not conducted, thus regarding commercially available apps, only those found in our database search were included in our review. We selected studies with mobile applications addressing psychological interventions for college students. A total of 2,158 participants were included in the 8 selected studies and most interventions were delivered through mobile apps only and based in cognitive behavioral therapy. Results suggested that college students accept and adhere to these interventions and preliminary evidence of efficacy was demonstrated in different disorders, such as stress, anxiety, depression and risky behaviors such as alcohol and tobacco abuse and sexual knowledge. We conclude that universities, particularly college counseling services, may benefit from mhealth interventions, not only to address college students' mental health but to decrease some of its difficulties related to lack of human resources. Specifically in covid-19 pandemic context, these interventions may contribute significantly by promoting and delivering psychological interventions at a safe distance.

## Introduction

Over the last decade numerous mental health mobile applications have been developed and made available for users (Bakker et al., 2016). Smartphones demonstrate numerous advantages such as great computing capacity, mobility, and more rapid and efficient access to information by using mobile applications (Donker et al., 2013). The enthusiasm of smartphones for healthcare initiatives led to the emergence of a novel field called mHealth (Ben-Zeev et al., 2014) defined as the use of mobile technologies to deliver or support psychological or mental health interventions and includes mobile devices such as smartphones, tablets, Personal Digital Assistants, and wearable devices (Clough and Casey, 2015b; Alyami et al., 2017). In clinical settings, mHealth may enhance face-to-face treatments, increase patient engagement in therapy sessions and adherence to therapy principles; provide better use of clinician time and resources and improve treatment outcome and risk of relapse (Clough and Casey, 2015b). Several studies have shown that mental health apps and cognitive behavioral therapy (CBT)-based apps are efficacious (Rathbone et al., 2017; Linardon et al., 2019). However, despite clinical potential, interest and early supporting evidence, one factor that seems to limit mental health apps is low engagement or poor adherence to the intervention (Torous et al., 2018).

One of the areas were mental health apps can have a significant impact is in universities. College years are a sensitive period to the onset of several mental health disorders (Kessler et al., 2007) and many studies have reported a significant rise in serious mental health illness among college students (Hunt and Eisenberg, 2010; Storrie et al., 2010; Auerbach et al., 2018). Major Depressive Disorder (MDD) and Generalized Anxiety Disorder (GAD) were identified as the most common disorders found in college students (Auerbach et al., 2018). University counseling services constitute a valuable resource to support college student mental health and wellness (Spooner, 2000) and a challenge that seems to be common across several counseling services is the growing student demand for these services and the limited resources to face these demands (Johnson and Kalkbrenner, 2017; Shaw et al., 2017; Auerbach et al., 2018; Lee and Jung, 2018). College students are also large consumers of technology and communicate frequently online (Shaw et al., 2017). A study by Wilansky et al. (2016) referred that mobile applications may increase youth adherence to Cognitive Behavioral Therapy (CBT) and improve treatment outcomes. Research suggests that mHealth is already being used to increase students' awareness and to deliver health-related interventions with increasing popularity; preliminary findings indicate that students are open and willing to use these interventions (Johnson and Kalkbrenner, 2017).

Mobile technologies for mental health assume an important role considering our current reality of pandemics resulting from covid-19 infectious disease. Covid-19 is an infectious disease cause by a coronavirus that rapidly expanded worldwide, and some of the protective measures include physical distancing, wearing a mask, avoiding crowds and close contact, and regularly cleaning your hands (World Health Organization, 2020). College students, alongside with children and health workers, are one of the most exposed groups to develop post-traumatic stress disorder, anxiety, depression and other symptoms of distress (Saladino et al., 2020). Studies conducted during covid-19 pandemic in China concluded that almost half of Chinese college students that participated in the study experienced anxiety symptoms (Fu et al., 2021) and are more likely to suffer from stress, anxiety and depression than the general population (Li et al., 2020). Several studies highlight the need to monitor students' mental health during the pandemic and the delivery of timely and appropriate interventions (Cao et al., 2020; Fu et al., 2021) such as the importance of technological devices or digital interventions (Saladino et al., 2020). Covid-19 brought several challenges to mental health services delivery, thus many therapists rapidly adhered to telehealth to replace in-person contact (Taylor et al., 2020). The same authors state that this disease presents an imperative for mental health services to make digital mental health interventions available in routine care and not only in response to covid-19 crisis.

Previous systematic reviews with college students and mobile interventions often explore a wide range of mHealth interventions and technology (e.g., Johnson and Kalkbrenner, 2017). Our review will focus on (1) mental health mobile applications that include a psychological intervention targeting a mental disorder, (2) college students, and (3) randomized controlled trials and acceptability and feasibility studies. We aim to explore how mobile apps are being developed to address college students' mental health in universities, if they accept and adhere to these interventions and if these interventions demonstrate efficacy. A search will be made for peer-reviewed articles of mental health mobile apps in scientific databases. The present review will not conduct a search in app stores mainly because acceptability and efficacy outcomes are not usually reported in app stores and because it would demand a different type of search strategy. Thus, in the current review we aim to review all published literature, in scientific databases, on psychological interventions using mobile applications, in the last 12 years, for college students. Our main objective is to review efficacy outcomes, through randomized controlled trials, of mobile app-based psychological interventions compared to traditional therapy or a waiting list control group in reducing psychological symptomatology among college students. Additionally, we intend to explore how mobile interventions are being accepted by college students and which conceptual frameworks are being used to develop these interventions. Considering the recent context of covid-19 pandemics, we aim to reflect on the impact and contributions of mHealth interventions for universities and college students.

## Methods

We used the search method of the Preferred Reporting Items for Systematic Reviews and Meta-Analyses (PRISMA) (Moher et al., 2009).

### Eligibility Criteria and Information Sources

Inclusion criteria considered (1) target population: college students; (2) types of intervention: psychological interventions delivered through mobile applications (self-guided); mobile applications combined with web-based interventions or mobile applications combined with face-to-face treatments; (3) Primary outcome measures that target specific psychological disorders or symptomatology (i.e., anxiety, depression, social anxiety, stress, PTSD, alcohol abuse); (4) clear report of the psychological intervention, specifying theoretical basis or treatment model and therapeutic techniques; (5) Types of studies: randomized controlled trials (RTC) or quasi-experimental designs that clearly report efficacy outcomes and feasibility and acceptability studies since they contribute with valuable information about conceptual framework and some provide preliminary effectiveness results; (6) written in English; (7) published in the selected scientific databases. Exclusion criteria consisted in (1) studies with young adults (not students); (2) mobile interventions based on text messages; (3a) mobile interventions targeting physical or medical conditions (e.g., diabetes, physical activity, nutrition, weight control etc.); (3b) studies about mobile learning apps (e.g., anatomy); (3c) studies about smartphone addiction; (4) internet and computerized based interventions; (5) study protocols.

Our main objective is to review conceptual framework, acceptability, and efficacy outcomes of mobile app interventions addressing mental health for college students. A search of mobile apps commercially available in the app store was not conducted in this review since, although important, demands a different type of search and selection process, and often don't report acceptability and efficacy results (in the app store). Thus, we considered that it would be more suited to do a review, with this group of apps, separately. A narrative approach was used for extraction and synthesis of the data. Studies were identified through three major electronic databases, namely, Web of Science, Pubmed, and Scopus. An update literature search was performed in January 2021 using the same information sources.

### Search and Study Selection Process

The following search keywords were considered “mobile interventions,” “smartphones,” “mobile application,” “mHealth,” “mobile technology,” “college students,” “students,” “university,” “campus.” Two authors independently conducted a thorough search in the three major scientific databases with the mentioned keywords, using primarily the combination “mobile interventions” AND “college students” with year filter between 2008 and 2019. A search update was performed in January 2021 with the same study selection process. In a first instance, studies including keywords in titles and/or abstracts were selected for further thorough review. After identifying eligible studies, duplicates were removed, and full papers were examined regarding eligibility criteria. A list of studies was produced by each author. Afterwards, both authors discussed their list of included studies, and by agreement, a final list of studies was produced.

### Data Extraction

Data extraction was performed by two independent researchers and included year of publication, demographic characteristics of participants, study design (RCT, quasi-experimental studies, single-arm pre-test post-test), study participants and interventions (i.e., population, conditions, sample size, outcome measures, mobile app characteristics, theoretical basis, and intervention modality), main results and findings.

### Assessment of Methodological Quality

The present review resorted to critical appraisal tools from the Joanna Briggs Institute for randomized controlled trials and quasi experimental studies (non-randomized experimental studies). The Checklist for Randomized Controlled Trials [The Joanna Briggs Institute (JBI), 2017a] was utilized to assess the methodological quality of the included RCTs and the Checklist for Quasi-Experimental Studies (non-randomized experimental studies) [The Joanna Briggs Institute (JBI), 2017b] to assess methodological quality of quasi-experimental studies and studies with a one group pre-test post-test design. Each study was assessed using JBI checklists for RCT or quasi-experimental studies.

## Results

### Study Selection

**Figure 1:**
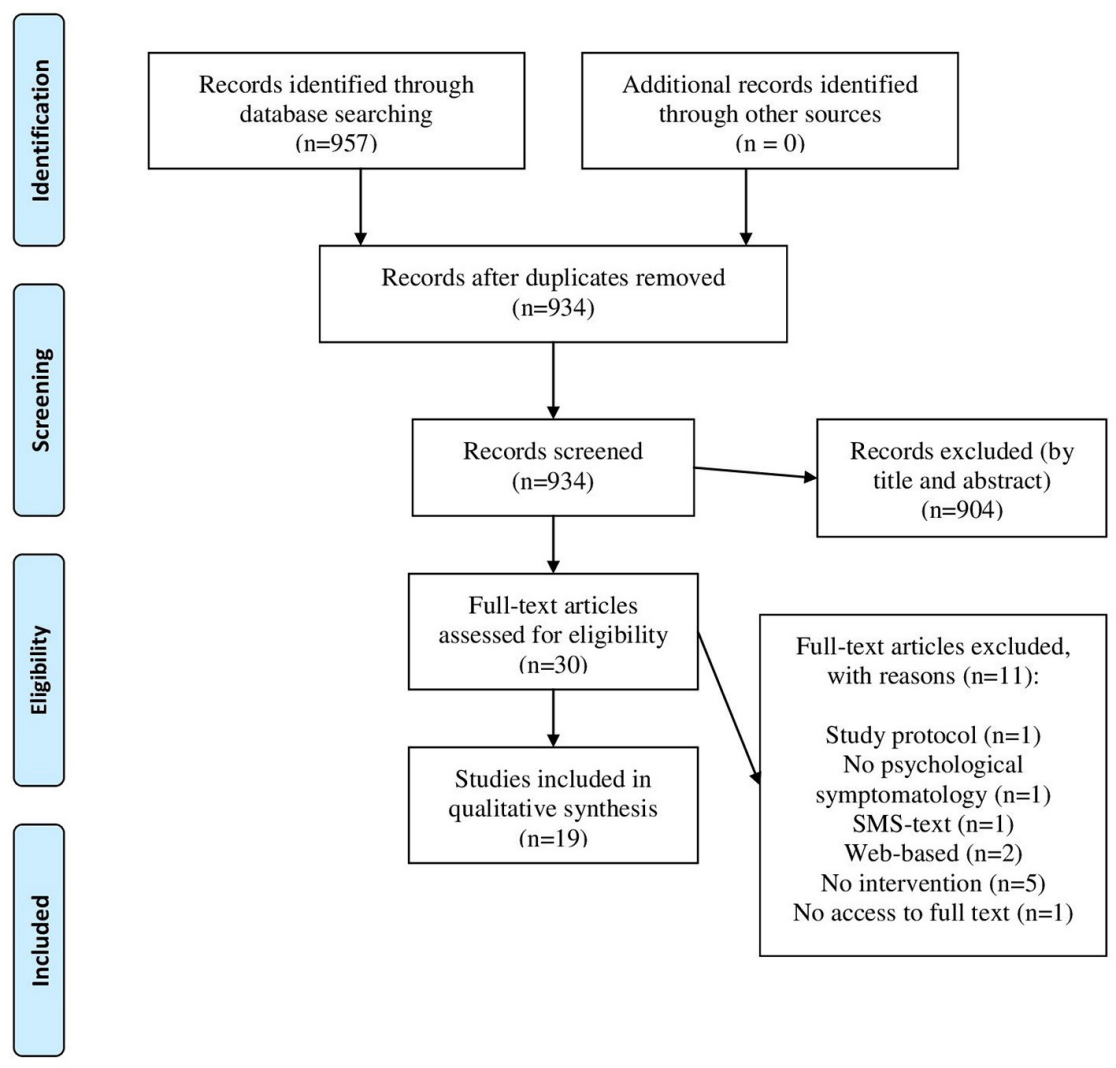
PRISMA flow diagram. From: Moher et al. (2009).

As we can see in Figure 1 our search identified 957 published articles. Afterwards, we removed 23 duplicates and a review of title and abstracts excluded 904 articles. A total of 30 full-text articles were assessed for eligibility, where 11 were excluded due to motives of being a study protocol, thus not presenting feasibility or efficacy outcomes; lack of a psychological intervention or a psychological disorder; being web-based intervention or having no access to article full text. A total of 19 studies were included and examined in accordance with inclusion criteria.

### Demographic Characteristics

A total of 3,399 college students were included in the selected studies (*n* = 19) for this systematic review. Eleven studies included college students with self-reported psychological symptomatology (i.e., elevated stress, generalized anxiety disorder (GAD), PTSD), two studies included first-year college students and the remaining studies included non-treatment seeking college students (*n* = 6). Most studies occurred in the USA (*n* = 12), others occurred in Germany (*n* = 1), Sweden (*n* = 1), Canada (*n* = 1), United Kingdom (UK) (*n* = 2), Australia (*n* = 1), and Iran (*n* = 1).

### Intervention Characteristics

Mobile intervention apps for college students target anxiety (*n* = 7), depression (*n* = 7), stress (*n* = 5), alcohol consumption and risky drinking (*n* = 4), smoking (*n* = 1), and sexual behaviors (*n* = 1), Post-traumatic stress disorder (PTSD) (*n* = 1). Table 1 resumes all further interventions characteristics.

**Table 1:** Mobile interventions characteristics.

**Study**	**Disorder**	**Intervention**	**Conceptual framework**	**Techniques**	**Human support**
*Nod* Bruehlman-Senecal et al. (2020)	Loneliness Depression	Mobile application Self-guided	Positive Psychology; Mindfulness-based self-compassion; Cognitive Behavioral Therapy (CBT)	CBT skill building exercises; social skills; cognitive restructuring; mood-rating tool	No
*BioBase* Ponzo et al. (2020)	Anxiety Depression	Mobile application + wearable band (BioBeam)Self-guided	CBT; mindfulness; biofeedback; behavioral activation theory	Psychoeducation; mood tracking via EMA; passive data collection (physical exercise, sleep quality and heart rate) obtained via BioBeam wearable band; respiration biofeedback (deep-breathing tool).	No
*Lantern* Newman et al. (2020)	Generalized Anxiety	Mobile application Self-guided	CBT; adapted from evidence-based psychotherapy program for GAD (Newman et al., 2020).	Psychoeducation; automatic thoughts; cognitive reframing; exposure; mindfulness.	Yes. Coach with various educational backgrounds (e.g., clinical psychology, marriage and family therapy, health coaching).
*Feel Stress Free* McCloud et al. (2020)	Anxiety Depression	Mobile application Self-guided	CBT	Behavioral relaxation activities (calm breathing, mindfulness-style meditation, deep muscle relaxation, self-hypnosis); mood tracking; thought challenging;	No
*Headspace* Flett et al. (2020)	Distress	Mobile application Self-guided	Mindfulness	Meditation; mindful breathing, body scan, sitting meditation, practice of non-judgement of thoughts.	No
*ACT daily* Haeger et al. (2020)	Anxiety Depression	Mobile application	Acceptance and Commitment Therapy (ACT);	Training on four ACT components: acceptance; cognitive defusion; present moment awareness; values connection; ecological momentary assessment (EMA)	No
*PTSD smartphone-app mindfulness* Reyes et al. (2020)	PTSD	Mobile application	ACT	Audio-guided mindfulness meditations; video lessons based on ACT principles;	No
*IntelliCare for College Students* Lattie et al. (2020)	Depression Anxiety	Mobile application	Eclectic (e.g., ACT, CBT, positive psychology, problem-solving therapy)	Mood rating and mood journal tool: allowed mood rating; calendar tool: history of their mood rating and journal entries; weekly symptom check: personalized feedback; short psychoeducational lessons; suite of interactive skill-focused IntelliCare apps	No
*Calm* Huberty et al. (2019)	Elevated Stress	Mobile application	Mindfulness meditation; Octalysis Framework; Self-determination theory (SDT);	Meditation; gamification.	No
*Headspace* Fish and Saul (2019)	Depression	Mobile application	Mindfulness	Guided mindfulness meditation	No
*Aramgar* Borjalilu et al. (2019)	Stress	Mobile application	Mindfulness-based stress reduction (MSBR);	Mindfulness skills (i.e., mindful practice, eating, breathing; body scan; managing thoughts; kindness practice);	No
*StudiCare Stress* Harrer et al. (2018)	Elevated Stress	Internet + mobile application	Web-based GET.ON Stress – Program; CBT; Lazarus transactional model of stress; Human Accountability Model	Problem-solving strategies; emotion regulation strategies; psychoeducation: Students' specific topics; homework assignments (app); self-monitoring (app); automatic daily messages containing short motivational prompts and ultrabrief training exercises;	Yes. Trained student in a master's program in Psychology (eCoach). Contact solely established online with no face-to-face meetings.
*DeStressify* Lee and Jung (2018)	Stress Anxiety Depression Sleep Quality of life.	Mobile application (commercially available app).	Mindfulness	Mindfulness exercises: grounding visualization, gratitude, imagining the life you want, and finding meaning;	No
*SmarTrek* Kazemi et al. (2018)	Risky drinking	Mobile application	Motivational interviewing; Ecological Momentary Intervention (EMI).	Self-monitoring; psychoeducation; incentives for behavioral changes; interactive Games; know your BAC (blood alcohol content); virtual coach: fully automated and sends daily text messages; personalized feedback;	No
*Mind the moment (MtM)* Leonard et al. (2017)	Alcohol consumption in female college students	Mobile application (with integrated sensorband for Electrodermal activity).	Ecological momentary intervention (EMI) integrated with a wearable sensorband; Motivational interviewing; CBT; Unified Theory of Use and Acceptance of Technology (UTUAT).	Personalized feedback;Emotion regulation strategies [e.g., controlled breathing, mindfulness meditation and individually-identified strategies (i.e., listening to music or exercise)].	Yes. 2 in-person brief counseling sessions with clinician.
*Telecoachapp* Gajecki et al. (2017)	Alcohol consumption	Mobile application (web-based).	CBT; Skills training to reduce excessive alcohol consumption;	Registration of alcohol consumption; relapse prevention skills; risk situation analyses or refusal exercises; relaxation exercises; positive thought exercises; urge surfing training.	No
*Therapist assisted online (TAO)* Benton et al. (2016)	Anxiety	Internet + mobile application	CBT	Interactive online educational modules based on CBT; Mindfulness exercises; Exposure exercises; weekly text messages for support and encouragement; homework assignments (app); summary of clients' activities and BMH-20 scores in dashboard screens (for therapists).	Yes. Weekly 10-12 min videoconference with a therapist.
*SEX101* Jackson et al. (2016)	Sexual Behaviors	Mobile application (Web-based)	Theory of Reasoned Action (TRA); Theoretical Model (TTM) of behavior Change	Four separate modules: condom use; contraceptive use; sexual partner relationships and alcohol use.Modules provided: general information, quizzes, brief scenario-based videos and comparison statistics on peer sexual norms to address attitudes and subjective norms for each behavior (condom use, contraceptive use etc.); skill building exercises (e.g., quizzes, games).	No
*Mobile feedback intervention for heavy drinking and smoking* Witkiewitz et al. (2014)	Heavy-episodic drinking (HED) and smoking	Mobile application	Brief Alcohol Screening and Intervention for College Students (BASICS); CBT; Mindfulness	Personalized feedback; feedback about smoking and “urge-surfing”; mindfulness-based relapse prevention.	No

We considered that most studies, with self-guided apps, focus on prevention (*n* = 15) and the studies that included human support (therapists and coaches) and a TAU group were more focused on a treatment approach (*n* = 4). However, many studies with self-guided apps, included students with elevated psychological symptomatology (i.e., elevated stress, diagnosed PTSD, or GAD), and it isn't always clear the nature of their intervention.

Intervention modality varied between a combination of internet and mobile app intervention (*n* = 2) and mobile app intervention only (*n* = 17), from these 17 studies, two apps were combined with a wearable band to permit passive data collection. When combining mobile apps with internet interventions, the mobile app functioned mostly as a tool offering support for homework assignment or working as a diary app by enabling monitoring of mood fluctuations or stress levels [e.g., Harrer et al. (2018)]. Human support was considered in 4 mobile apps (Lantern; TAO; StudiCare Stress; Mind the Moment), two mobile interventions included therapists and two included a coach, StudiCare Stress app included a trained master's student in Psychology (named an eCoach) and Lantern app included a coach with various educational backgrounds. Human support varied from weekly 10–12 min brief videoconferences, to 2 face to face sessions and online sessions only.

Regarding conceptual framework most researchers used CBT intervention or CBT third wave techniques to conceptualize these apps (*n* = 17). Most CBT apps include mindfulness exercises (*n* = 11), some are solely based on mindfulness (*n* = 4) or acceptance and commitment therapy (ACT) (*n* = 2). One mobile app is focused on CBT and a biofeedback intervention (BioBase app). Some used CBT intervention as a part of a larger program such as GET.ON Stress, a stress management program, adapted to college students; or BASICS, an alcohol intervention program for college students. In some cases, CBT was combined with other psychological models such as Lazarus Transactional Model of Stress (GET.ON Stress program) or the Unified Theory of Use and Acceptance of Technology (UTAUT). The StudiCare Stress app also included an adherence-focused guidance concept according to the human accountability model. Only two studies did not resort to CBT, the SmarTrek app that used motivational interviewing and the SEX101 that used two psychological models, the Theory of Reasoned Action (TRA; Fishbein and Ajzen, 1975) and the Transtheoretical Model (TTM) of behavior Change (Prochaska and DiClemente, 1984). Additionally, SmartTreak and MtM added an Ecological Momentary Intervention (EMI) and Witkiewitz et al. (2014), BioBase app and ACT daily included an Ecological Momentary Assessment (EMA).

As for specific techniques more than half of the mobile apps include mindfulness exercises; other included psychoeducation or general information about the target disorder; include data collection self-monitoring; exposure; systematic desensitization and relaxation exercises. Other features refer to quizzes and interactive games; virtual coach; passive sensing through sensorband; all apps for risky drinking and excessive smoking included personalized feedback on drinking patterns and motives for drinking, feedback includes information about smoking and “urge-surfing” or strategies to increase student's emotional awareness. All apps were designed to provide education, collect data, monitor/track behavior, some provide personalized feedback or guidance in CBT exercises (in some cases homework assignments).

Few studies gave information regarding privacy and security. For example, Benton et al. (2016) referred that TAO security and privacy included authentication, password protection, and encryption of databases and Lee and Jung (2018) stated that data was collected and stored on secure systems and accessed through computers with password protection and encryption.

### Methodological Quality

Tables 2, 3 resumes the methodological characteristics of the included studies. Eleven studies are randomized controlled trials (RCT) (Witkiewitz et al., 2014; Gajecki et al., 2017; Harrer et al., 2018; Lee and Jung, 2018; Fish and Saul, 2019; Huberty et al., 2019; Bruehlman-Senecal et al., 2020; Flett et al., 2020; McCloud et al., 2020; Newman et al., 2020; Ponzo et al., 2020) and two studies are considered quasi-experimental trials (Benton et al., 2016; Borjalilu et al., 2019). Four studies considered a single-arm pre-test-post-test study design (Jackson et al., 2016; Leonard et al., 2017; Haeger et al., 2020; Lattie et al., 2020; Reyes et al., 2020) and one study included two groups through an iterative process (Kazemi et al., 2018).

**Table 2:** Methodological characteristics.

**Study**	**Research design**	**Comparator**	**Participants**	**Primary outcome measures**	**Intervention duration**	**Main results**	**Main findings**
*Nod* Bruehlman-Senecal et al. (2020)	RCT	IG vs. WLG	*N =* 221 First-year college students	Loneliness UCLA-8; Generalized Anxiety Disorder Scale (GAD-7); Patient Health Questionnaire (PHQ-9).	4 weeks Follow-up at week 8. 4 assessment points: baseline; week 2; week 4; week 8.	Significant condition-by-baseline loneliness interaction to predict week-4 depression (*p =* 0.2, Np^2^ = 0.02) and sleep quality (*p* = 0.004, Np^2^ = 0.04)	Nod presented benefits for first-year college students with elevated risk (e.g., loneliness and depression) by buffering from heightened mid-semester loneliness and depression.
*BioBase* Ponzo et al. (2020)	RCT	IG vs. WLG	*N =* 262; Students with elevated anxiety and stress	State-Trait Anxiety Inventory-short version – 6 item (STAI-S-6);	4 weeks Follow-up at week 6 4 assessment points: baseline, week 2; week 4; week 6.	Significantly reduced anxiety at week 4 (*p =* < 0.001, *d* = 67), with sustained effects at a 2-week follow-up (*p =* < 0.001, *d* = 0.81); Increased perceived well-being at week 4 (*p* = 0.001; *d* = 0.65) and follow-up (*p =* < 0.001, *d* = 1.16); Significant reduction in depression was found at week 4 (*p =* < 0.001, *d* = 0.99)	There is evidence to support the efficacy of BioBase in reducing anxiety and increase perceived well-being in university students.
*Lantern* Newman et al. (2020)	RCT	IG vs. No treatment control group	*N =* 100 Students with self-reported GAD.	Generalized Anxiety Disorder Questionnaire for DSM-IV (GAD-Q-IV); Depression Anxiety Stress Scale (DASS stress)	3 months Follow-up at 9 months 3 assessment points: baseline; 3 month and 9 month.	Reduction on the DASS stress (*d* = 0.408); Greater probability of remission from GAD (*d* = 0.114); From those who remitted at post-treatment, 78.6% remained remitted.	Preliminary support of efficacy of a smartphone-based guided and self-help intervention for the treatment of some GAD symptoms in college students.
*Feel Stress Free* McCloud et al. (2020)	RCT	IG vs. WTG	*N =* 168 Students with anxiety and/or depression symptoms	Hospital Anxiety and Depression Scale – Anxiety subscale (HADS-A) and Depression subscale (HADS-D);	4 weeks Follow-up at 6 weeks 4 assessment points: baseline; week 2; week 4 and week 6.	Week 6: reduced depression symptoms (*p =* 00.6; *d* = 0.39); Week 4: reduced depression symptoms (*p* = 0.04; *d* = 0.27); reduced anxiety symptoms (*p* = 0.001; *d* = 0.58).	Feel Stress Free app demonstrates preliminary evidence of effectiveness in reducing symptoms of anxiety and depression.
*Headspace* Flett et al. (2020)	RCT	IG vs. WLG	*N =* 250 First year students	Kessler Psychological Distress Scale (K10);	3 months; 3 assessment points: beginning of semester 1; beginning of semester 2; end of academic year.	Weak evidence support of improvements in psychological distress over time. Participants in the IG who used the app more frequently reported improvements in psychological distress (−5 points, *R*^2^ change = 0.12) and college adjustment (+10 points, *R*^2^ change = 0.09).	Headspace app was associated with small improvement in distress and college adjustment.
*ACT daily* Haeger et al. (2020)	Single-arm pre- post-test design	IG	*N =* 11 Students suffering from anxiety and depression on the waiting list of college counseling centers (CCC)	Depression, Anxiety and Stress Scale (DASS);	2 weeks 2 assessment points: baseline and week 2.	Significant improvements in depression (*g* = 1.08), anxiety (*g* = 0.73), stress (*g* = 0.81), psychological flexibility (*g* = 0.64) (all *p* < 0.01). System usability ratings were within “excellent” range (*M* =9 0, SD = 0.66)	Results support that ACT daily is acceptable and usable as a self-guided intervention for depressed and anxious students waiting for therapy in CCC.
*IntelliCare* Lattie et al. (2020)	Single-arm pre-test-post-test design	IG	*N =* 20 College students with and without elevated symptoms of depression and anxiety	Patient Health Questionnaire (PHQ-9); Generalized Anxiety Disorder questionnaire (GAD-7); Qualitative user feedback;	8 weeks 3 assessment points: baseline, week 4 and week 8.	Significant improvements in Anxiety literacy (*p* = 0.045) and in the frequency with which participants used both cognitive (*p* = 0.04) and behavioral (*p* = 0.03) coping skills. High retention rate; High degree of usability.	IntelliCare app for college students was considered usable and engaging.
*PTSD smartphone-app mindfulness* Reyes et al. (2020)	Single-arm pre-test-post-test design	IG	*N =* 23 Veteran students with PTSD symptoms	Connor-Davidson resilience scale (CD-RISC); PTSD checklist for DSM-5 (PCM-5);	4 weeks 3 assessment points: baseline, week 2 and week 4.	Significant increase in mean resilience (*p* < 0.05) and mindfulness scores (*p* < 0.001) and decrease in experiential avoidance, PTSD and rumination scores across assessment time points (all *p* < 0.001). High levels of intervention satisfaction and usability;	Significant changes in resilience, mindfulness, PTSD, experiential avoidance and rumination.
*Calm* Huberty et al. (2019)	RCT	IG vs. WLG	*N =* 88 Students with perceived elevated stress.	Perceived Stress Scale (PSS-10); Mindfulness; Self-compassion;	8 weeks Follow-up at 12 weeks 3 assessment points: baseline, week 8 and week 12.	Significant differences in all outcomes: stress, mindfulness and self-compassion (all *p* < 0.04). Effects persisted at follow-up (all *p* < 0.03). Effect sizes ranged from moderate (*d* = 0.59) to large (*d* = 1.24).	Mindfulness meditation delivered through Calm app is effective in reducing stress and improving mindfulness and self-compassion in college students suffering from elevated stress.
*Headspace* Fish and Saul (2019)	RCT	IG vs. WLG	*N =* 72 College students	Patient Health Questionnaire (PHQ-9);	2 weeks 2 assessment points: baseline and week 2.	Significant interaction of group by time for depression (*p =* 0.021) Within-subjects (*p =* < 0.001) and between-group analyses (*p =* 0.008) showed a significant decrease in depression severity scores.	A gamified mindfulness meditation app significantly decreased depression symptom severity among college students.
*Aramgar* Borjalilu et al. (2019)	Quasi-experimental trial	IG vs. face-to-face therapy +app vs. face-to-face therapy only	*N =* 68; College students	Depression, anxiety and stress scale (DASS-21)	6 weeks 2 assessment points: baseline and week 6.	Significant difference in the mean reduction of depression, anxiety and stress between conditions (all *p* < 0.001). Group 2 (Blended intervention) had the greatest mean score reduction on stress, depression and anxiety among the three groups.	Results suggested that intervention through the blended therapy was more influential on mental health (stress, depression and anxiety) compared with the other two groups.
*StudiCare Stress* Harrer et al. (2018)	RCT	IG vs. WLGBoth conditions had full access to treatment as usual (TAU).	*N =* 150 College students with elevated levels of stress	Perceived Stress Scale (PSS-4);	7 weeks Follow up at 3 months after baseline. 3 assessment points: baseline, week 7 and 3 month.	Significant effects of the intervention compared with the waitlist control group for stress (*d* = 0.69), anxiety (*d* = 0.6) and other outcomes after post-treatment. Effects were sustained at follow-up.	Internet and mobile-based interventions may be an effective approach to reduce symptoms of stress and other health and college related outcomes, as well as symptoms of depression.
*DeStressify* Lee and Jung (2018)	RCT	IG vs. WLG	*N =* 163; College students	Perceived Stress Scale (PSS-10); Stait Trait Anxiety Inventory (STAI); Quick Inventory of Depressive Symptomatology Self-Report (QIDS-SR);	4 weeks 2 assessment points: baseline and week 4.	Reduced trait anxiety *(p* = 0.17, np^2^ = 0.01), and improve general health (*p* = 0.001, np^2^ = 0.07), energy (*p* = 0.01, np^2^ = 0.05), emotional well-being (*p = 0.0*1, np^2^ = 0.05).	Mindfulness-based apps may be an effective alternative to support university's student's mental health.
*SmartTrek* Kazemi et al. (2018)	2 Groups run sequentially through theater testing.	Single-arm pre-post-test design	*N =* 10 College students	The readiness ruler; Daily Drinking Questionnaire (DDQ); Usefulness, Satisfaction and Ease of Use (USE).	1 week for Group 1 2 weeks for Group 2 2 assessment points: baseline and post intervention.	6 in 10 participants reported that the app had a positive effect on their drinking less. Good app usability. Games were considered to be the best feature and Daily log, Coach and Personalized Feedback as the most useful features.	Most of the participants agreed that SmarTrek was easy to use and the information provided was useful and had a positive effect on decreasing their drinking.
*TeleCoach app* Gajecki et al. (2017)	3-arm RCT	Assessment-only control group vs. IG vs. WLG	*N =* 330; Students with excessive alcohol consumption	Daily Drinking Questionnaire (DDQ); The Alcohol Use Disorders Identification Test (AUDIT);	6 weeks 3 assessment points: baseline; week 6 and week 12.	Proportion of students with excessive alcohol consumption declined in both intervention and wait list group compared to controls at first (*p* < 0.001) and second follow-ups (*p =* 0.054). Reductions in the intervention group in quantity of drinking at first follow-up (*p =* 0.037) and in frequency of drinking at both follow-ups (*p =* 0.034).	The app demonstrated potential for reducing excessive alcohol use among college students.
*Mind the Moment (MtM)* Leonard et al. (2017)	Single-arm pre-test-post-test design.	IG	*N =* 10 Non-treatment seeking college students with risky drinking	Alcohol Use Disorders Identification Test – Consumption (AUDIT-C) Physiological measures: Electrodermal Activity (EDA) through sensorband.	3–4 weeks 2 assessment points: baseline and 3–4 week.	High levels of acceptability. Qualitative findings indicate that sensorband-elicited alerts promoted an increase in awareness of thoughts, feelings and behaviors related to environmental stressors and drinking behaviors.	These interventions have great potential to individualize behavioral interventions to reduce problem drinking and other health behaviors.
*Therapist assisted online (TAO)* Benton et al. (2016)	2-arm non-randomized controlled trial	IG vs. TAU	*N =* 1,241 Students with moderate levels of anxiety	Global health measure (GHM)	7 weeks Assessment points: baseline and 7 weekly assessments.	TAO scores were significantly greater than treatment-as-usual scores. Improvements across time were significantly greater for TAO than treatment-as-usual participants. Effect sizes range from small (LF *d* = 016, GMH and well-being *d* = 0.20), to moderate (anxiety *d* = 0.31).	This study indicates that TAO may be an effective treatment for anxiety disorders with a positive influence on an overburdened practitioner and treatment center.
*SEX101* Jackson et al. (2016)	Single-arm pre-test-post-test design	IG	*N =* 118_–_College students	Sexual health knowledge (developed by the researchers) to assess knowledge.	1 week Follow-up at 3 months after intervention completion. 2 assessment points: baseline and follow-up at 3 month.	Ninety-six percent (*N =* 114) of the participants showed an increase in contraceptive use knowledge from pre-test to post-test (*p* = 0.013). There was no statistically significant change in intention to reduce sexual risk behaviors or actual risk reduction. Most participants (93.9%) were very satisfied or satisfied with the intervention program suggesting good acceptability.	A brief and theory-driven mobile app intervention to decrease sexual risk behaviors among college students may be effective in increasing knowledge and attitudes about contraceptive use.
*Mobile feedback intervention for heavy drinking and smoking* Witkiewitz et al. (2014)	3-arm RCT	IG vs. daily monitoring vs. minimal assessment control.	*N =* 94 Non-treatment seeking college students	Daily Drinking questionnaire (DDQ); Daily Smoking Questionnaire (DSQ); Young Adult Alcohol Problems Screening Test (YAAPST); Ecological Momentary Assessment (EMA) Measures.	14 days Ecological momentary assessment (EMA) (monitoring period) Follow-up assessment 1 month after the monitoring period.	At 1-month follow-up there were significant reductions in number of cigarettes per smoking day in both the mobile intervention (*d* = 0.55) and mobile assessment conditions (*d* = 0.45). Mobile intervention group showed lower likelihood of any drinking during the intervention.	Results provide initial evidence that mobile assessment could be effective in reducing smoking among college students. It also provides initial data supporting feasibility and acceptability of the mobile intervention.

**Table 3:** JBI Checklist for randomized controlled trials.

**JBI Checklist**	**Studies**
Randomization	11/11
Allocation to treatment groups concealed	3/11
Treatment groups similar at baseline	10/11
Participants blind to treatment assignment	0/11
Those delivering treatment blind to treatment assignment	1/11
Outcome assessors blind to treatment assignment	0/11
Treatment groups treated identically other than the intervention of interest	10/11
Follow-up complete, and if not, were differences adequately described and analyzed	9/11
Participants were analyzed in the groups in which they were randomized	6/11
Outcomes measured in the same way for treatment groups	11/11
Outcomes measured in a reliable way	11/11
Appropriate statistical analysis	11/11
Appropriate trial design, and any deviates from the standard RCT accounted for in the conduct and analysis of the trial	11/11

Eleven of the included studies are RCTs and the total sample size ranges from 72 to 330 college student participants; the overall duration of the intervention range from 14 days to 3 months and when we consider follow-ups, the longest trial lasted for 9 months. Most RCTs included as a control group a waiting list control trial (*n* = 8). Following JBI critical appraisal tool, we consider that all RCTs reported that participants were randomly assigned to treatment groups, 9 out of 11 studies provided detailed description of the randomization procedure and two studies merely stated that the participants were randomly assigned. As for allocation concealment, three studies provided information about allocation concealment. For example, Harrer et al. (2018) stated that the randomization process was performed by a researcher not involved in the study, and although they weren't able to blind participants to study conditions, during the randomization process, they were able to conceal the allocation from participants, researchers, and e-coches. Ten studies provided information and reported similar groups at baseline. As for blinding participants, or those delivering treatment and even outcome assessors to treatment conditions may be difficult and even unachievable in this type of studies; several studies reported this issue, pointing to the inability to blind their participants to treatment conditions. There were incomplete follow-ups; however they were generally adequately described and analyzed. Six RCTs provided detailed information about intention-to-treat analyses (ITT); the remaining studies excluded participants, lost to follow-up, from analysis. All studies used primary outcome measures with good validity and reliability. The large majority of RCTs also included quantitative and/or qualitative self-report measures to evaluate usability, acceptability, user satisfaction, or app adherence.

The studies by Benton et al. (2016) and Borjalilu et al. (2019) were considered as quasi-experimental studies. The first study included a large sample size (*n* = 1,241) with overall duration of the intervention of 7 weeks. They included a wait-list treatment as usual control group and the intervention group received the intervention of study. The primary outcome measure was adequately validated and provided multiple measurements along the intervention as well as pre and post assessment. Differences between groups in terms of follow-up were adequately described and analyzed. This study presented many missing data and the linear mixed-effects models was utilized to estimate parameters for missing values. As for Borjalilu et al. (2019), they conducted a study with three conditions and 68 college students, who were randomly assigned into the three groups, but no further detailed information was given about the randomization process. There were pre- a post-assessments and follow up was complete. Outcomes were measured in a reliable way and participants, from both groups, were assessed in the same way.

In this review there is a significant number of a single group pre-test-post-test design studies that aimed to evaluate acceptability and feasibility; only one study (Jackson et al., 2016) aimed to evaluate efficacy with this design. Sample sizes were similar between studies, ranging from *n* = 10 to *n* = 23, with overall duration (intervention) of 3–4 weeks. Adequate and validated main outcome measures were used. The SEX101 (Jackson et al., 2016) had a larger sample size compared to the previous studies and a follow-up assessment of 3 months after intervention completion. However, the overall duration of the intervention was very small (pre-test and intervention had to be complete in 1 week and it takes 40 min to complete) and some outcome measures were developed by the researchers with few information regarding reliability.

### Intervention Outcomes and Effect Sizes

A study conducted by Newman et al. (2020) assessed the efficacy of Lantern, a self-help mobile app to treat generalized anxiety disorder. Study results demonstrated a significant reduction on the DASS stress scores (*d* = 0.408) and greater probability of remission from GAD (*d* = 0.114). Lantern revealed moderate effects in reducing anxiety, stress, and depression. BioBase is a biofeedback self-guided mobile app combined with wearable device (BioBeam), to treat anxiety in college students. Ponzo et al. (2020) conducted a RCT to assess BioBase efficacy and results indicated that a 4-week intervention significantly reduced anxiety (*d* = 0.67), depression (d = 0.99), and increased perceived well-being (*d* = 0.65) demonstrating moderate to large effects. Sustained large effects at 2-week follow-up was found for anxiety (*d* = 0.81) and perceived well-being (*d* = 1.16).

McCloud et al. (2020) conducted a RCT to assess efficacy of Feel Stress Free app for the treatment of depression and anxiety symptoms. Results showed that there was a significant reduction of depression symptoms at week 4 (*d* = 0.27) and week 6 (*d* = 0.39), and significant reduction of anxiety symptoms at week 4 (*d* = 0.58). Overall effect sizes ranged from small to moderate.

Bruehlman-Senecal et al. (2020) studied Nod, a mobile app designed to reduce loneliness during the transition to college. Their RCT results indicated significant condition-by-baseline loneliness interaction to predict week-4 depression (Np^2^ = 0.02) and sleep quality (Np^2^ = 0.04), suggesting that Nod buffered participants with higher baseline loneliness against heightened midquarter depression and poor sleep quality. Calm, is a mindfulness-based app, and its efficacy was tested among students with elevated stress. The study results of Huberty et al. (2019) found significant differences among conditions in all outcomes, namely, significant reduction in perceived stress (*d* = 1.24), significant improvements in mindfulness (*d* = 1.11), and self-compassion (*d* = 0.84).

Harrer et al. (2018) conducted a randomized controlled trial to evaluate the efficacy of Studicare Stress, a stress management intervention app for college students. Their results indicated significant effects of the intervention compared with the waitlist control group for stress at post-test (*d* = 0.69) and at 3-month follow-up, other secondary outcome measures also yielded significant effects such as anxiety (*d* = 0.76), depression (*d* = 0.63), college related productivity (*d* = 0.33), and academic work impairment (*d* = 0.34). Thus, Studicare Stress revealed moderate to large intergroup effects for the reduction of perceived stress and other health and college related outcomes.

Lee and Jung (2018) conducted a pilot study to evaluate efficacy of DeStressify, a mindfulness-based app on stress, anxiety, depressive symptomatology, sleep behavior, and other variables. Results indicated that when using the app during 4 weeks, students in the experimental group at post-test reported less trait anxiety (${\text{N}}_{\text{p}}^{2}$ = 0.040); an improve in several quality of life subscales, such as general health, that significantly differed between treatment condition in post-intervention scores (${\text{N}}_{\text{p}}^{2}$ = 0.07). A significant difference was also found in energy or fatigue subscale between treatment conditions (${\text{N}}_{\text{p}}^{2}$ = 0.05). An interaction effect was found in the emotional well-being subscale (${\text{N}}_{\text{p}}^{2}$ = 0.05). The author interpreted the partial eta squared values of 0.0099, 0.0588, and 0.1379 as small, medium, and large effect, respectively, following suggestions by Cohen (Field, 2009). This indicates that we can verify small (trait anxiety) to medium effects for general health, energy or fatigue and emotional well-being.

Telecoach app (Gajecki et al., 2017) was evaluated using a 3-arm randomized controlled trial and results demonstrated that the proportion of students with excessive alcohol consumption declined in both intervention and wait list control group compared to controls at first and second follow-ups. Secondary analysis showed reductions for the intervention group in quantity of drinking at first follow up and in frequency of drinking at both follow-ups. Across both follow-ups the odds ratios for not having excessive weekly alcohol consumption in the intervention group (1.95) was almost twice as high as for controls (1.00). Secondary analysis by gender showed that the odds ratio for not having excessive alcohol consumption among men in the intervention group compared to male controls was higher (2.68) than women in the intervention group (1.71) compared to women controls.

Witkiewitz et al. (2014) conducted a 3-arm randomized controlled trial to evaluate a mobile feedback intervention for heavy-episodic drinking (HED) and smoking among college students, and they concluded that at 1-month follow-up there were significant reduction in number of cigarettes per smoking day in both the mobile intervention (*d* = 0.55) and mobile assessment conditions (*d* = 0.45) with moderate effects. No significant results were observed on HED or concurrent smoking and drinking. As for Benton et al. (2016) quasi-experimental study, the intervention group showed improvements across time significantly greater than treatment as usual participants, for all primary outcomes except Life Functioning (LF) subscale. The size of these effects ranged from small (*d* = 0.16) for LF, Global Mental Health and Well-Being (d = 0.20) to medium for Anxiety (*d* = 0.31).

### Usability, Acceptability, and Feasibility Outcomes

The large majority of the included studies evaluated acceptability and students' satisfaction with the intervention. From the 19 studies, eight studies explored adherence/satisfaction and six used adequately valid scales or methods to assess usability or satisfaction with app use. Some studies also used metrics obtained through the mobile app (*n* = 2). Most studies, created their own items to assess satisfaction with the intervention. Overall, we could observe good retention rates across studies, however as Gajecki et al. (2017) specifically noted in there study, there is a possibility that their fairly high retention levels could result from the desire of some participants to win an iPad (reward to participate in the study) with no actual intention to use the app. Out of the 19 studies, 10 gave rewards to their participants.

All studies that evaluated satisfaction reported moderate to high client satisfaction with the intervention. The MtM app (Leonard et al., 2017) demonstrated that 60% of the participants reported “mostly” or “very” satisfied with the sensorband and 50% with the mobile app. Also, 93.9% of the participants were very satisfied or satisfied with the intervention program of SEX101 app (Jackson et al., 2016). However, this particular study produced large attrition rates (50%) and as the authors of this study noted information regarding app components that need to be improved, added or removed should be collected. In the Witkiewitz et al. (2014) EMA app, over 65% of the participants reported an increase in awareness of their drinking and/or smoking and 60% stated that they would recommend this study to a friend because it provided greater awareness and they could help a friend reduce their drinking and/or smoking. Kazemi et al. (2018) demonstrated good usability of SmartTrek and the best feature reported by students was “Games” and the most useful features was “know your BAC” and “My strategies” that monitored alcohol intake, created behavioral change plans and reminded them of their goals. None of the studies, that provided human support (therapists), explored acceptability and satisfaction of the therapist with the intervention.

### Implications and Contributions of mHealth Interventions for College Students in Covid-19 Context

Covid-19 infectious disease emerged in China and rapidly expanded around the globe, leading to an unexpected pandemic, which completely changed our daily lives and significantly limited physical and social contact with significant repercussions to our physical and mental health. Specifically in college students that live in a constant and thriving social interaction, covid-19 pandemic had a strong negative impact on mental health and may have contributed to the increase of several preexisting barriers and limitations to college counseling services. Considering these restraints, mHealth interventions may play an important role in a pandemic context due to its ubiquitous, remote and innovative functionalities that may facilitate access to evidence base treatments for mental health and also, its provider and facilitator (therapist).

Taking into consideration the included studies and their characteristics, acceptability, satisfaction and efficacy outcomes, we may determine that these interventions can significantly contribute in several important aspects related to college students' mental health. To our understanding, mobile app technologies may significantly contribute to promote mental health in college students targeting several specific disorders, such as anxiety, stress, depression, smoking, and alcohol abuse. It is also attainable to support students with coping strategies for elevated stress, anxiety, smoking, and alcohol abuse. Through mobile technologies, therapists may monitor and keep track of their patients' symptomatology and well-being, check homework assignments, and contact their patients' regularly through chat or messages, remotely. Overall, mobile technologies provide spontaneous and remote access to app content whenever we want, particularly in the comfort of our home. It helps us maintain physical distance from mental health professionals and counseling services without interrupting treatment.

## Discussion

### Summary of Evidence

Our search for studies addressing mobile health apps for college students in university settings gathered 19 studies with different conceptual frameworks and study designs. In this review we could verify an increase in studies using mobile interventions for college students over the years, particularly in the last year, which may indicate an increasing trend in mobile use for the delivery of health interventions for college students. The large majority of studies are being developed in North America and Europe.

Regarding target disorders we can verify that most apps target anxiety, depression and stress, others target risky or excessive drinking, PTSD and sexual behaviors. Overall, mobile interventions showed promising results to reduce psychological symptomatology associated with stress, depression, anxiety and general student's mental health. As for drinking, smoking, and sexual behaviors, the included apps seemed to reduce excessive drinking and smoking and increase contraceptive use and knowledge but not the intention to reduce sexual risk behaviors or actual risk reduction. Most of the mobile interventions showed medium to large effect sizes for the main variables the app was designed to intervene, which may indicate that these interventions are well conceptualized and grounded according to the best available empirical evidence. Some of the included studies aimed to evaluate acceptability and feasibility and overall, these apps demonstrate good acceptability and feasibility among college students, supporting the hypothesis that students may accept and adhere to these interventions.

When we explore conceptual frameworks of these mobile apps we verify that many studies adopted CBT as the main intervention, particularly Mindfulness exercises. Effectively, CBT is well-established and particularly known as an effective treatment for several mental health disorders, and have demonstrated its efficacy when delivered through apps (Rathbone et al., 2017). In some studies the intervention was complemented with psychological models, which have been shown to increase intervention efficacy (Webb et al., 2010). Aside from psychological models/theories for behavioral change, one study incorporated a technological model, namely the Unified Theory of Use and Acceptance of Technology (UTUAT). There seems to be a strong application of psychological models and intervention techniques, indicating that there is a concern in adequately conceptualizing these interventions following evidence base principles. However, considering that we are studying mobile health interventions with significant emphasis in technology, very few studies incorporated technological models. Also, security and privacy features are also rarely mentioned and increasingly relevant in this type of interventions, best practices should be known and shared, reflecting in a mobile app quality indicator.

Regarding therapist role in mobile interventions, only 4 studies incorporated human support, two studies included therapists and two studies included a trained psychology student. From the mentioned studies, one used the human accountability model to inform this support. We consider that even though most of these apps intend to reduce therapist time and subsequently reduce therapist caseload and overburdened, this process may be optimized and better conceptualized using human support models. Moreover, evidence shows that app based interventions with therapist support has shown to produce larger effects (Linardon et al., 2019).

As for methodological quality of the included studies, most studies aimed to evaluate efficacy and resorted to a randomized controlled trial, which is natural since RCTs are known as the golden standard to evaluate efficacy. All trials randomly assigned their participants to treatment conditions; however the number of studies that performed randomization concealment and blinding was almost non-existent. This reflects the difficulty of concealment and blinding in these type of studies and the limitation of the RCT study design when assessing efficacy in this type of interventions. Most studies also use a waiting list control group; given that many studies included students with elevated psychological symptomatology (that have to wait weeks/months to get access to the intervention) and the difficulty of blinding participants with this type of comparator we wonder if this is the best control group to use in this studies. Other research designs are also being explored in these studies and should be considered, so we can obtain efficacy results timelier and reliably (Clough and Casey, 2015a). Many studies adopted a pre-test post-test study design in order to evaluate acceptability and feasibility, even though this research design is considered a weak experimental study design, we consider that for the purpose and objectives of the studies this design was well-applied. Good overall retention rates may indicate treatment feasibility and acceptability. However, most studies were of short duration, with small samples and in controlled settings, with the addition of significant rewards. Additionally, many outcome measures were self-reported and not always congruent with app adherence rates. User metrics (e.g., how many times a participant accessed the app) provided by mobile apps may contribute to more accurate indicators of use and adherence to the intervention. Also, qualitative studies exploring perceived usefulness and user experience with the app intervention may also contribute to understand and overcome some barriers of adherence and engagement. Rewards are sometimes our best option to find participants, however when we are studying acceptability and adherence to these interventions, rewards may produce biased results. Recent studies opted to reward outcome measures completion, rather than app use.

A final question that emerged while exploring the studies is associated with the limited visual content of the apps included in the studies. Few studies included images/visual content of the mobile apps; some studies reported how they developed the app but provided little information about app design. A study by Torous et al. (2018) concluded that most mhealth apps suffer from low engagement and adherence and this may be, along with other issues, due to poor usability and because most apps are not user-friendly. It is important that researchers provide more frequently studies regarding user's needs and report multidisciplinary teams when building (native) apps, since this area often needs involvement of psychologists, software engineers, and designers/interaction designers. Also these tools, in clinical settings (e.g., counseling services), should be designed and optimized regarding all end users: students and therapists. Therapists' point of view and evaluation was often forgotten in the included studies that involved therapists.

Mobile apps may be customized and designed under practically unlimited possibilities. They can be developed to promote, prevent or intervene in a specific mental health disorder; to promote well-being and to deliver treatment under different levels of therapist support in different mental health services. Thus, they can be implemented and tailored according to specific needs. It is important to continue studying these interventions using user-centered designs and rigorous efficacy and effectiveness studies. We consider that universities, including college counseling services, may benefit from mhealth interventions, not only to address college student mental health but to decrease some of its difficulties related to few human resources. In a context of quarantine and confinement at home, where physical and social distance is imperative, these interventions assume special importance. They facilitate mental health promotion and support therapist and patient contact at a safe distance, avoiding treatment interruption.

### Limitations

The current review presents a major limitation since we limited our search scope to the mentioned databases. Registered clinical trials and commercially available apps in app stores were not included, thus we may have missed already developed or apps that are being currently studied for college students. We may have failed to identify studies with relevant information regarding the application of mHealth intervention in college settings when we didn't consider “young adults,” since it may not include college students or occur in college settings.

## Conclusion

The current systematic review shows that mobile apps for mental health intervention in college students exists and demonstrates good acceptability and feasibility. They also demonstrate efficacy among students. Overall we may conclude that mHealth interventions may turn out to be a great resource and tool to implement in counseling services, offering therapists and students many advantages. Particularly in the current pandemic context, these interventions demonstrate innumerous possibilities and promising solution to address college students' mental health and overcome many barriers associated with treatment access.

Future studies addressing mobile apps in college students, should invest in user-centered design studies so we can better understand what students and therapists (also attending university counseling services workflow) value more in a mobile based psychological intervention, to better adapt and tailor the intervention to user's needs. Effectively, acceptability and feasibility results among therapists are lacking in studies that use mobile intervention with therapist support. Future investigations should also explore diversity when developing and studying future apps, examining the applicability and efficacy of other theories/models. Also, we consider that studies should describe the development process of the mobile application (e.g., by including visual content) so we can better understand what is actually being evaluated and how it may impact efficacy results, in terms of usability and design. Lastly, students are large consumers of technology and so it may be important to invest more in these interventions, doing larger studies with more students, with superior methodological quality and avoiding large monetary rewards.

## Data Availability Statement

The original contributions presented in the study are included in the article/supplementary material, further inquiries can be directed to the corresponding author.

## Author Contributions

CO searched for studies to include in the systematic review and wrote sections of the manuscript. AP and PV revised the manuscript and contributed to the conception of the study. CN, JG, and BA contributed to organize data extraction and the search of studies in the scientific databases. All authors contributed to the manuscript revision, read, and approved the submitted version.

## Conflict of Interest

The authors declare that the research was conducted in the absence of any commercial or financial relationships that could be construed as a potential conflict of interest.
